# A Synchronized Spin Model for Black-Hole Accretion Systems

**DOI:** 10.3390/e28060663

**Published:** 2026-06-10

**Authors:** Masahiro Morikawa, Akika Nakamichi

**Affiliations:** 1 RIKEN, Wako 351-0198, Saitama, Japan; 2 Department of Physics, Ochanomizu University, Bunkyo 112-8610, Tokyo, Japan; 3 General Education, Kyoto Sangyo University, Kyoto 603-8555, Kyoto, Japan; nakamichi@cc.kyoto-su.ac.jp

**Keywords:** black-hole accretion, synchronized spin model, collective dynamics, amplitude modulation, magnetic reconnection

## Abstract

Black-hole accretion systems exhibit a characteristic coexistence of activities: broad-band X-ray variability, hot coronae, wide-angle winds, and both steady and discrete jets. This coexistence suggests a persistently time-dependent magnetic background in which noisy fluctuations and explosive release are both essential. In this paper, we connect them all to the storage, organization, and intermittent reconnection-mediated release of magnetic energy, and we propose a Synchronized Spin Model (SSM) in which multiple local dynamos in a rotating accretion flow are represented as interacting macro-spins. Their synchronization, partial synchronization, excursion, and reversal define a compact set of collective variables that organize both timing statistics and large-scale morphology. In this picture, multiscale magnetic reconnection converts stored magnetic energy into coronal heating, flares, intermittent outflows, and discrete jet activity, while the same synchronization dynamics produce amplitude modulation and demodulation, providing a route to 1/*f*-like variability, rms–flux/Taylor-like scaling, and approximately log-normal statistics of the demodulated envelope. We further argue that, although the continuous flux distribution in black-hole systems is more naturally discussed in multiplicative or log-normal terms, broader event-catalog statistics remain useful for describing suitably defined burst hierarchies, particularly by analogy with solar and stellar flare systems. The hard/soft cycle of X-ray binaries is then interpreted as motion through magnetic state space.

## 1. Introduction

Black-hole accretion systems show a recurring combination of phenomena: broad-band X-ray variability, hot coronae, winds, compact jets, and transient ejecta. In X-ray binaries (XRBs), these observables reorganize across the hard, intermediate, and soft states; in active galactic nuclei (AGN), analogous spectral and outflow changes occur on much longer timescales, including in changing-look AGN [1,2,3,4,5]. The main theoretical challenge is therefore not to explain any one component in isolation but to identify a compact physical language that links timing, topology, and outflow morphology.

Existing approaches address important subsets of the problem. Propagating-fluctuation models reproduce red-noise continua and multiplicative variability [6,7,8]. Ordered-flux models explain how jets and winds are launched once a suitable magnetic geometry exists [9,10]. Reconnection and plasmoid pictures explain impulsive dissipation, flares, and intermittent ejections [11,12,13]. What remains less clear is why these observables reorganize together.

We address this problem with a Synchronized Spin Model (SSM). The model is not intended as a microscopic alternative to General Relativistic Magnetohydrodynamics (GRMHD). Instead, it is a mesoscopic effective description in which the inner accretion flow is coarse-grained into interacting magnetic domains, or “macro-spins.” Their alignment, partial synchronization, excursion, and reversal define a small set of collective variables that organize both the timing statistics and the magnetic topology of the flow.

We emphasize at the outset that the SSM is not intended to replace viscous accretion evolution, SOC-like avalanche pictures, or propagating-fluctuation models. These frameworks naturally describe how mass supply, disk stresses, and inward propagation of fluctuations can produce broad-band timing behavior and set the slow background evolution of the accretion flow. The role of the SSM is complementary. It introduces a mesoscopic magnetic layer between the accretion cascade and local plasma dissipation: accretion supplies energy and boundary conditions, magnetic fields store and organize part of that energy into coherent structures, and reconnection rapidly converts the stored magnetic energy into coronal heating, flares, winds, and steady or transient jet channels. Thus, the slow clock of the state evolution is not identified with the elementary local reconnection time, but with the buildup, synchronization, and reconfiguration of magnetic coherence.

This paper proceeds as follows. Section 2 summarizes the observational constraints. They motivate us to introduce the coarse-grained spin variables and the Synchronized Spin Model (SSM) in Section 3. We then discuss the statistical consequences of spin synchronization in Section 4. The SSM maps the magnetic-state variables to coronae, flares, winds, and jets, as shown in Section 5. Section 6 describes how the SSM reproduces the observed statistics. Section 7 connects the model to XRB cycles and the q-diagram. Section 8 describes observational falsifiability of the SSM scenario. Section 9 discusses the SSM’s niche within existing frameworks, its limitations, and possible validation routes. Finally, Section 10 concludes this paper.

## 2. Observational Constraints from Black-Hole Systems

A unified magnetic-state model must account for at least six empirical regularities.

(i) Broad-band variability

**Figure 1:**
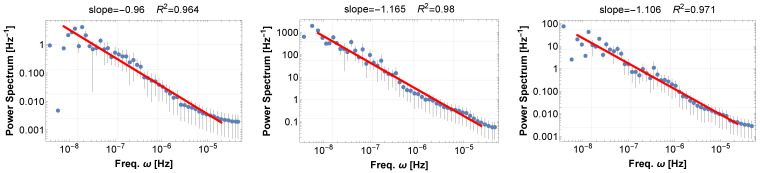
Representative power spectral densities (PSD) derived from 16 yr MAXI X-ray light curves for an AGN (Mrk 421; **left**), a black-hole X-ray binary (Cyg X-1; **center**), and a neutron-star X-ray binary (1A 1742-294; **right**). Approximately one-third of the several hundred MAXI sources examined show pink broad-band variability across part of the band. This figure is obtained as follows. The original MAXI X-ray flux data are linearly interpolated to produce a time series with equal time intervals. After the PSD is calculated using the DFT, the data are averaged within each octave (blue dots), with error bars. Then, we obtain the sliding-fit window that maximizes the coefficient of determination $R^{2}$: the red line is this best-fit range. The procedure and the scheme are the same for all the PSD graphs in this paper.

Many accreting black-hole systems show power spectral densities (PSDs) of the form

(1) $$P\left(f\right)\propto f^{\alpha },\;\alpha ∼-1,$$

where P(f) is the Fourier power at temporal frequency *f* and α is the effective low-frequency slope. This behavior persists across part of the band, although the slope is state-dependent and not universal [6,7,14]. Figure 1 illustrates representative MAXI power spectral density (PSD) for an AGN, a black-hole XRB, and a neutron-star XRB. The presence of 1/*f* variability in both black-hole and neutron-star accretors argues that the long-memory component is primarily a property of the accretion disk and not of the central compact objects.

(ii) Continuous-flux statistics and event-catalog statistics

We distinguish two related but non-identical statistical levels.

First, for the continuous X-ray light curve, many accreting black-hole systems are more naturally characterized by multiplicative variability, a linear rms–flux relation, and an approximately log-normal flux distribution than by a single scale-free law for the instantaneous flux itself [7,14,15]. A useful phenomenological representation is therefore

(2) $$p\left(F\right)=\frac{1}{F\;\sigma_{\ln F}\sqrt{2\pi }}\exp\;\left[-\frac{{\left(\ln F-\mu_{\ln F}\right)}^{2}}{2\sigma_{\ln F}^{2}}\right],$$

where *F* is the flux and $\mu_{\ln F}$ and $\sigma_{\ln F}$ are the mean and standard deviation of lnF. This form is the observationally better-motivated default for the continuous light-curve statistics of black-hole accretion systems.

Second, if one decomposes the variability into threshold-defined shots, flares, or ejecta, the resulting event catalog can, in some systems or analyses, exhibit broad, approximately scale-free energy or amplitude statistics,

(3) $$N\left(E\right)\propto E^{-\alpha_{E}},$$

where N(E) is the occurrence rate of events with energy or amplitude proxy *E* and $\alpha_{E}$ is the corresponding index. Such power-law-like event statistics are particularly common in solar and stellar flare catalogs [11,16], whereas in black-hole systems, the inferred event distribution depends more strongly on how events are defined and should not be conflated with the flux distribution of the continuous light curve [7].

(iii) Steady and transient jets

In black-hole X-ray binaries, compact steady jets are typically associated with the hard state and can persist throughout a substantial part of this state. By contrast, transient or discrete ejecta are preferentially observed during the hard-to-soft transition [1,3]. This motivates a decomposition into a quasi-steady and a transient channel,

(4) $$P_{\mathrm{jet}}^{\mathrm{steady}},\;P_{\mathrm{jet}}^{\mathrm{disc}},$$

where the superscripts denote steady compact-jet power and discrete ejection power. The two channels need not respond identically to magnetic order or state change.

(iv) Persistent coronae

Coronal emission is long-lived yet highly variable. A useful coarse-grained description is

(5) $$L_{\mathrm{cor}}∼\int_{V}Q_{\mathrm{diss}}\;dV,\;Q_{\mathrm{diss}}∼\eta_{\mathrm{eff}}J^{2}+Q_{\mathrm{turb}}+Q_{\mathrm{rec}},$$

where $L_{\mathrm{cor}}$ is the coronal luminosity, *V* is the emitting volume, $Q_{\mathrm{diss}}$ is the local dissipation rate, *J* is the current density, $\eta_{\mathrm{eff}}$ is an effective resistivity, and $Q_{\mathrm{turb}}$ and $Q_{\mathrm{rec}}$ denote unresolved turbulent and reconnection heating. The point of Equation (5) is not precision modeling but to emphasize that the corona can be sustained by many small dissipative sites rather than by a single coherent event [12,17].

(v) Structured winds

AGN and disk winds are often modeled as continuous outflows, but observations increasingly show clumpy or intermittent structure [18,19,20]. It is therefore useful to separate a continuous background from a discrete magnetic release,

(6) $$P_{\mathrm{wind}}=P_{\mathrm{wind}}^{\mathrm{cont}}+P_{\mathrm{wind}}^{\mathrm{disc}},$$

where $P_{\mathrm{wind}}^{\mathrm{cont}}$ and $P_{\mathrm{wind}}^{\mathrm{disc}}$ denote continuous and discrete wind power, respectively.

(vi) State transitions and excursions

In XRBs, hardness, timing properties, jet behavior, and broad outflow signatures reorganize together along the HID/q-diagram [2]. In AGN, changing-look events indicate that large spectral changes need not be accompanied by equally strong new jets [4,5]. Any successful model must therefore distinguish full large-scale magnetic reorganization from more limited excursions in magnetic state space.

These constraints motivate a nonequilibrium magnetic description in which timing statistics and outflow morphology arise from the same evolving collective variables.

## 3. Physical Basis of the Synchronized Spin Model (SSM)

We now describe our simple model, starting with the basic features common to all astronomical accretion-disk systems.

### 3.1. From Rotating MHD to Macro-Spin

The starting point is a rotating, conducting, magnetized flow described at the continuum level by resistive MHD,

(7) $$\rho \left(\frac{\partial u}{\partial t}+u\cdot \nabla u\right)=-\nabla p+\rho g+\frac{1}{4\pi }\left(\nabla \times B\right)\times B+\nabla \cdot \Pi -2\rho \;\Omega \times u,$$

with ∇·B=0. Here, ρ is mass density, u is velocity, *p* is pressure, g is gravitational acceleration, B is magnetic field, Π is viscous stress, Ω is the rotation vector. In a rotationally constrained regime,

(8) $$Ro=\frac{U}{2\Omega L}\ll 1,$$

where *U* and *L* are representative velocity and length scales. When Ro≪1, the dominant Coriolis force (the last term in Equation (7)) is balanced by the pressure gradient (the first term): −∇p=2ρΩ×u. Taking the curl of this balance yields

(9) Ω·∇u=0

i.e., the rotation can maintain columnar structures aligned with the rotational axis even in a turbulent flow. This motivates a Taylor-column-like picture of locally coherent dynamo domains, used here only as a coarse-grained prototype for the inner disk. For each coarse-grained domain $V_{i}$, we define a magnetic moment-like variable

(10) $$S_{i}\equiv \frac{1}{V_{i}}\int_{V_{i}}r\times J\;dV,\;J=\frac{c}{4\pi }\nabla \times B,$$

where r is the position vector measured from the domain center, J is current density, and *c* is the speed of light. The quantity $S_{i}$ is not the literal spin of a particle. It is a coarse-grained magnetic moment that measures the net handedness of current circulation within domain $V_{i}$. We then normalize it to define a macro-spin,

(11) $$s_{i}=\frac{S_{i}}{|S_{i}|},\;\left|s_{i}\right|=1.$$

**Figure 2:**
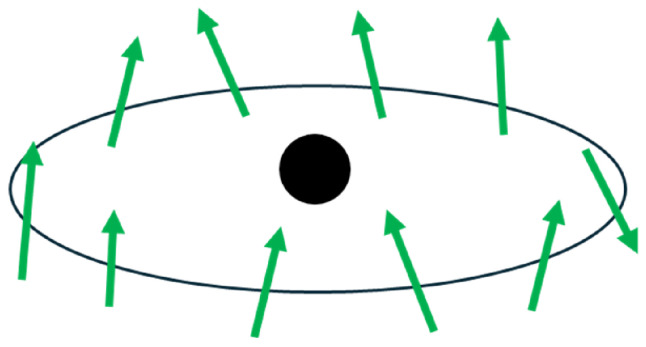
Schematic configuration of the SSM local dynamo domains (green) embedded in the inner part of the accretion disk. Each spin represents a coarse-grained magnetic domain or the winding current around a locally coherent column-like structure. Their collective alignment, frustration, and reversal determine the large-scale magnetic topology and its observable consequences.

The ensemble $\left\{s_{i}\right\}$ is the basic mesoscopic state variable of the SSM. Figure 2 shows the intended geometry: the spins occupy the inner part of the disk and represent coarse-grained magnetic dynamo domains rather than individual field lines.

### 3.2. Effective Interactions and Collective Dynamics

We now set up the evolution equation of the macro-spins defined above. The spin equation should be understood as a mesoscopic effective equation, not as a closed-form derivation from the full MHD equations. The full MHD variables contain a large number of turbulent, intermittent, and reconnection-driven degrees of freedom. Neighboring macro-spin domains interact through induction, flux constraints, and magnetic stress transmission.

A minimal Lagrangian for the macro-spin variables $\left\{s_{i}\right\}$ can be written, almost uniquely, by requiring rotational invariance [21]. The Lagrangian of a fundamental theory should be a scalar under spatial rotations, and an effective Lagrangian inherits the same symmetry constraints. We therefore retain the lowest-order scalar terms in the spin variables.

(12) $$\begin{array}{cc} L & =K-V, \end{array}$$

(13) $$\begin{array}{cc} K & =\frac{1}{2}\sum_{i=1}^{N}{\dot{s}}_{i}^{\;2}, \end{array}$$

(14) $$\begin{array}{cc} V & =\mu \sum_{i=1}^{N}{\left(\Omega \cdot s_{i}\right)}^{2}+\frac{\lambda }{2N}\sum_{i<j}s_{i}\cdot s_{j}, \end{array}$$

where *N* is the number of macro-spins, *K* is an inertial kinetic term for slow collective motion, and *V* is the effective interaction potential.

The size of the coarse-grained domain is determined by the magnetic and hydrodynamic correlation lengths, the disk scale height, and the degree of magnetic arrest. Thus, the number N of macro-spins is not a fundamental constant; it is an effective resolution parameter determined by the ratio between the active disk volume and the correlation volume of each local dynamo domain.

The coefficient μ weights alignment with the rotation axis, while λ measures the mean spin–spin coupling.

The signs of the effective couplings are chosen so that the ordered spin state is energetically favored. This choice is not meant to represent a microscopic exchange interaction but rather a coarse-grained consequence of rotationally biased local dynamo action. Each Taylor-column-like eddy winds electric currents around its axis and thereby produces a dipolar magnetic moment. Since these columns are embedded in the same differentially rotating disk, the sign of their helicity and current circulation is not random but biased by the common angular velocity and shear. Neighboring columns with parallel magnetic moments can be connected by smoother magnetic field lines, whereas antiparallel moments tend to create current sheets and reconnection sites. Therefore, at the level of the effective spin Hamiltonian, the interaction is ferromagnetic, corresponding to λ<0. Similarly, μ<0 represents the tendency of local spin moments to align with the rotation of the system, either parallel or anti-parallel.

Equation (14) is not derived from first-principles GRMHD. It is the lowest-order rotationally symmetric closure that favors either axis alignment or collective ordering and therefore serves as a reduced state model.

The present SSM is a conservative system, in contrast to real accretion flows, which are highly dissipative. Despite being conservative, the SSM is fully chaotic and highly random, capturing some important aspects of the irregular collective behavior seen in dissipative systems. In fact, the dissipative version of SSM [22] yields qualitatively similar magnetic activity.

To see the origin of the representative evolution equation, write each spin in polar form relative to Ω and consider the angular degree of freedom $\theta_{i}$. Variation of the Lagrangian then gives, schematically,

(15) $${\ddot{\theta }}_{i}=-\mu \Omega^{2}\sin2\theta_{i}-\frac{\lambda }{N}\sum_{j}\sin\left(\theta_{i}-\theta_{j}\right).$$

The first term comes from the derivative of ${\left(\Omega \cdot s_{i}\right)}^{2}\propto \cos^{2}\theta_{i}$ and therefore restores spins toward the rotation axis; either parallel or anti-parallel. The second term is the usual phase-coupling term obtained by differentiating pairwise dot products $s_{i}\cdot s_{j}=\cos\left(\theta_{i}-\theta_{j}\right)$. Equation (15) is therefore a second-order synchronization equation closely related to the inertial Kuramoto model [23,24]. In the present paper, it is used only as a reduced synchronization language, not as a microscopic derivation from MHD.

### 3.3. Order Variables

For synchronization theory, the physically useful morphological variable is the vector magnetization,

(16) $$M=\frac{1}{N}\sum_{i=1}^{N}s_{i},\;M=\left|M\right|.$$

Large *M* corresponds to a comparatively ordered, nearly dipolar state; small *M* corresponds to a disordered or multipolar state.

To characterize reversal-prone boundaries, we define the reversal-gradient activity

(17) $$G=\int_{V}{\left|\nabla M\left(x,t\right)\right|}^{2}\;dV,$$

which is large when neighboring regions carry strongly different local magnetizations. To distinguish closed from open topology, we define the open-flux fraction

(18) $$f_{\mathrm{open}}=\frac{\Phi_{\mathrm{open}}}{\Phi_{\mathrm{tot}}},$$

where $\Phi_{\mathrm{open}}$ is the magnetic flux threading field lines that escape the local system and $\Phi_{\mathrm{tot}}$ is the total unsigned flux. A fourth useful quantity is the axial alignment factor

(19) $$C_{\mathrm{ax}}=\frac{M\cdot \Omega }{\left|M|\;|\Omega \right|},$$

which measures how well the ordered component aligns with the rotation axis. The set $\left(M,G,f_{\mathrm{open}},C_{\mathrm{ax}}\right)$ will organize the phenomenology below.

**Figure 3:**
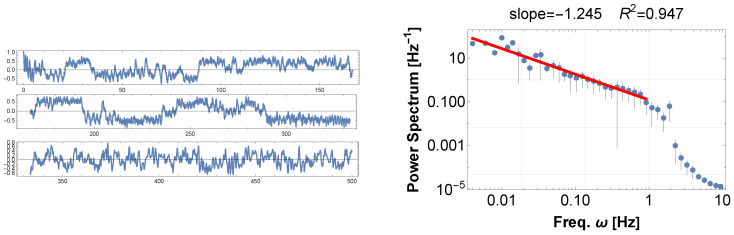
A typical SSM calculation result. The parameters are: $\mu =-10,\;\lambda =-20,\;N=9,\;K=-V/2,\;t_{\max}=500$. **Left**: time series of the projection of the mean spin onto the rotation axis, $C_{ax}$. The three stacked panels represent successive time segments of a single continuous run. Long intervals of one polarity are interrupted by rapid reversals; near-failures of reversal correspond to excursion-like events. **Right**: power spectral density (PSD) of the same series.

**Figure 4:**
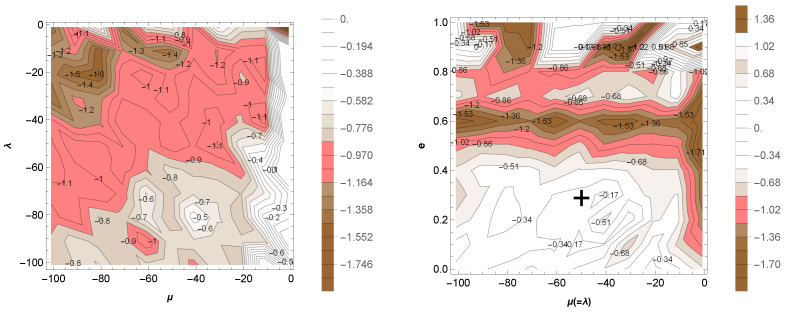
Distribution of the PSD power-law index in SSM parameter space. **Left**: map in the (μ,λ) plane, with K=−V/2, where the pink-noise regime occupies a broad region, indicating that the pink noise is not a critical phenomenon. **Right**: map in the plane of μ(=λ) and the initial energy in the form of e=1+(K/V). All the initial spins are almost up, and the potential energy is close to its minimum. Thus, e=1 means almost no initial kinetic energy, and e=0 means mild initial kinetic energy. The pink region becomes narrower in this representation, motivating the more detailed analysis in Figure 5, where the point marked “+” is examined.

**Figure 5:**
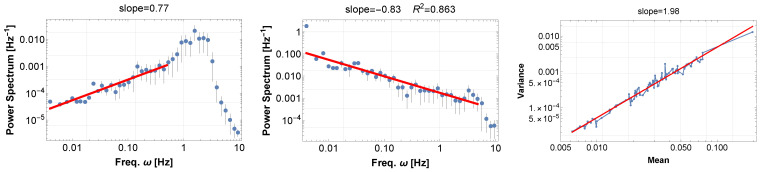
Statistical diagnostics for a representative parameter choice (μ=−50, λ=−50, N=10, e=0.3, time=500 (the point marked “+” in Figure 4)) from the pale/white-noise-like region in the right panel of Figure 4. **Left**: PSD of the detrended SSM data, which is not pink. **Middle**: PSD of the absolute value of the detrended data, which becomes pink, consistent with a demodulated amplitude envelope. **Right**: Taylor’s law test. The raw and merely detrended data do not follow Taylor’s law, whereas the absolute value of the detrended series does.

**Figure 6:**
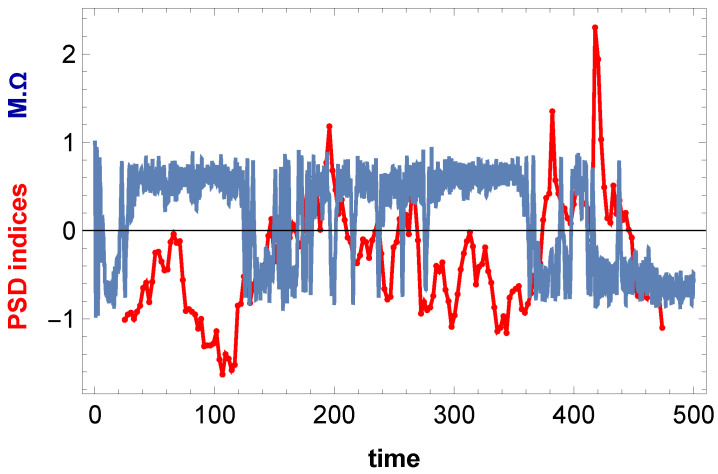
The spin magnetic parameter $C_{ax}$ (blue) and the running PSD indices (red) for the SSM calculation with the same parameter as for Figure 3, but another run. The running PSD indices are computed in overlapping windows whose length is 10% of the full record and whose start times are shifted by 1% each step. The PSD index tends to increase during excursions or polarity reversals, suggesting that global desynchronization weakens frequency crowding and therefore suppresses pink-noise behavior.

The numerical behavior of the model is illustrated in Figure 3, Figure 4, Figure 5 and Figure 6. We now explain them very briefly. The details will be described in the following sections. Figure 3 shows the time series of the projection of the mean spin onto the rotation axis and its PSD. Long polarity epochs are interrupted by rapid reversals, while smaller excursion-like events occur without a full sign change. Figure 4 maps the PSD slope over parameter space and shows that pink-noise-like behavior occupies a broad region. Figure 5 demonstrates that for some parameter choices the raw detrended series is not pink, whereas the absolute value of the detrended series becomes pink and satisfies Taylor’s law, consistent with an amplitude-modulation/demodulation interpretation. Figure 6 shows that the local PSD index tends to increase during polarity reversals, suggesting that strong global disorder suppresses frequency crowding at those times.

### 3.4. Reversal, Excursion, and Reconnection

In a finite-*N* SSM, the polarity branches +M and −M should be regarded as metastable. Since the full system is conservative but strongly chaotic, the unresolved internal degrees of freedom act as an effective finite-size bath for the collective magnetization. As a result, polarity reversals can happen as a result of chaos-assisted transitions between two metastable branches. In this sense, polarity reversal in SSM is a natural consequence of finite-*N* nonlinear collective dynamics. Only in the formal N→∞ limit would one expect a truly frozen spontaneous-symmetry-broken branch.

The model distinguishes a full global reversal,

(20) M→−M,

from a large excursion in which *M* is strongly disturbed but does not settle into the opposite polarity. This distinction will later be important for comparing XRB transitions with changing-look AGN.

Magnetic reconnection enters the model as a multiscale energy-conversion channel. A dimensional estimate for the released power is

(21) $$P_{\mathrm{MR}}∼\int_{A_{\mathrm{cs}}}\frac{B^{2}}{4\pi }v_{\mathrm{in}}\;dA,\;v_{\mathrm{in}}=\epsilon_{\mathrm{rec}}v_{A},$$

where $A_{\mathrm{cs}}$ is the total reconnecting current-sheet area, $B^{2}/4\pi$ is the magnetic energy density, $v_{\mathrm{in}}$ is the inflow speed into the sheet, $\epsilon_{\mathrm{rec}}$ is the dimensionless reconnection rate, and $v_{A}$ is the local Alfvén speed. Here, $v_{\mathrm{in}}$ is normalized by the local Alfvén speed, not because the inflow itself is an Alfvén wave, but because the reconnection exhaust is accelerated by magnetic tension to a speed of order $v_{A}$, and the inflow speed is a fraction of that characteristic MHD response speed. The factor $B^{2}v_{\mathrm{in}}/4\pi$ is the incoming Poynting-flux scale into the sheet, so Equation (21) estimates how rapidly magnetic energy can be converted into plasma heating, bulk acceleration, and non-thermal particles [12,13].

It is important to distinguish the local reconnection time from the longer mesoscopic and macroscopic magnetic-state timescales. The elementary reconnection time of a local current sheet is short, of order the local Alfvénic or dynamical response time. By contrast, the formation time of a reconnecting magnetic configuration, the waiting time between large events, and the lifetime of a coherent magnetic domain can be much longer. In the SSM, reconnection is therefore not the slow clock that sets the entire hard/soft cycle. It is the rapid conversion process that operates once magnetic energy has been accumulated and organized by the slower evolution of the magnetic state. This distinction allows fast local reconnection physics to participate in much longer state-dependent phenomenology without equating the reconnection time with the viscous time.

## 4. Statistical Structure of the SSM

### 4.1. From Synchronization to 1/f-like Variability

The central statistical idea is that dynamic synchronization produces long envelopes through frequency crowding and beating. In previous work, we argued that such envelope formation, followed by nonlinear demodulation, naturally produces pink-noise-like behavior [25]. The present SSM exhibits exactly that structure, which is why synchronization is central to the model.

Two nearby frequencies generate a beat envelope when ω≫Δω:

(22) cos[(ω+Δω)t]+cos[(ω−Δω)t]=2cos(Δωt)cos(ωt),

where ω is the fast carrier frequency and Δω is the beat frequency. Thus, a synchronized ensemble with many small frequency differences automatically produces low-frequency modulation.

If the collective signal is written as

(23) $$X\left(t\right)=\sum_{k}A_{k}\cos\left(\omega_{k}t+\phi_{k}\right),$$

with amplitudes $A_{k}$, phases $\phi_{k}$, and clustered frequencies $\omega_{k}$, then partial synchronization generates a hierarchy of envelope timescales. When the hierarchy of cluster sizes is broad, the envelopes naturally populate many timescales and produce pink continua rather than a single preferred period. In particular, if the spacing of the dominant modulation scales is approximately exponential, the demodulated envelope approaches a 1/f spectrum [25]. This is why the absolute value or square of the detrended signal is the relevant quantity in Figure 5 and later in Figure 9.

This amplitude-modulation mechanism for 1/*f*-like variability does not require that all of the observed low-frequency structure be attributed to a pre-existing hierarchy of intrinsic timescales. On the other hand, most existing explanations rely on a broad hierarchy of intrinsic timescales across the observed band [6,7,8].

Equivalently, the same phenomenology can be described in terms of a broad distribution of modulation, relaxation, or waiting times associated with magnetic coherence. For example, a superposition of relaxation modes,

(24) $$P\left(f\right)=\int D\left(\tau \right)\frac{2\tau }{1+{\left(2\pi f\tau \right)}^{2}}\;d\tau ,$$

produces P(f)∝1/f over a finite frequency range when D(τ)∝1/τ over the corresponding interval. We do not use this argument to claim a unique inversion from the PSD to D(τ). Rather, the observed 1/*f*-like behavior is taken as evidence for a broad, approximately scale-free hierarchy of coherence and waiting times. These timescales are distinct from the elementary reconnection time of a local current sheet.

### 4.2. Taylor’s Law and the Rms–Flux Relation

A scaling analogous to Taylor’s law follows naturally from the amplitude-modulation picture. In Equation (22), the high-frequency carrier cos(ωt) is modulated by the slowly varying factor cos(Δωt). In the same spirit, we model the short-time increment of any observed quantity $x_{t}$ as

(25) $$dx_{t}=A_{t}\;d\xi_{t},$$

where $A_{t}$ is a slowly varying envelope signal and $d\xi_{t}$ is a faster carrier or noise component without any structure. Within a time window *T* short enough that $A_{t}$ is approximately constant, the local variance becomes

(26) $${\mathrm{Var}}_{T}\left(dx\right)∼{\langle {\left(dx\right)}^{2}\rangle }_{T}=A_{t}^{2}{\langle {\left(d\xi_{t}\right)}^{2}\rangle }_{T},$$

while the local mean amplitude of the demodulated signal scales as

(27) $$\mu_{T}∼{\langle |dx|\rangle }_{T}=A_{t}{\langle |d\xi_{t}|\rangle }_{T}.$$

If the short-time statistics of $d\xi_{t}$ are approximately stationary within the window, then both ${\langle {\left(d\xi_{t}\right)}^{2}\rangle }_{T}$ and ${\langle |d\xi_{t}|\rangle }_{T}$ are effectively constant there, so eliminating $A_{t}$ between Equations (26) and (27) yields

(28) $${\mathrm{Var}}_{T}\left(dx\right)\propto \mu_{T}^{2}.$$

This gives a natural Taylor-like fluctuation scaling for the demodulated series. Here, the “mean” quantity is the local mean amplitude of the demodulated signal rather than the signed mean increment itself. When this variable *x* represents the energy flux *F*, this makes the connection to Taylor-like scaling explicit in astrophysical variability studies.

In black-hole accretion variability, the same phenomenon is usually discussed as the rms–flux relation rather than under the name of Taylor’s law [7,14]:

(29) $$\sigma_{\mathrm{rms}}=k\;\left(\bar{F}-C\right),$$

where $\bar{F}$ is the average flux over that time interval, $\sigma_{\mathrm{rms}}$ is the absolute root-mean-square (rms) variability amplitude within that interval, *C* is the trend, *k* is a constant. If we express this in terms of variance ($=\sigma_{\mathrm{rms}}^{2}$), this is equivalent to Taylor’s law, Equation (28). The present point is that the demodulated envelope can show the same quadratic scaling without separately postulating a wide deterministic hierarchy of low frequencies. The demodulation behavior seen in Figure 5 supports this viewpoint.

### 4.3. From Amplitude Modulation/Demodulation to Log-Normal Variability and Power-Law Tails

Section 4.2 presented a minimal phenomenological route from amplitude modulation/demodulation to multiplicative variability. Here, we recast the same idea in a form that is more consistent with the conservative SSM, and that also prepares the reduced stochastic description used again in Section 8.

The full SSM is conservative, so its total Hamiltonian is constant. However, after separating a slow collective phase from fast internal degrees of freedom,

(30) $$\theta_{i}\left(t\right)=\Phi \left(t\right)+\delta_{i}\left(t\right),$$

the collective sector can exchange energy with the chaotic internal sector $\left\{\delta_{i}\right\}$. Therefore, the relevant stochastic variable is not the conserved total energy of the full SSM but the partial energy assigned to the slow collective mode,

(31) $$E\equiv E_{\Phi }=\frac{1}{2}{\dot{\Phi }}^{2}+\frac{1}{2}\omega_{0}^{2}\Phi^{2}.$$

Near a locally stable branch, coarse-graining of the fast chaotic variables yields an effective second-order Langevin equation for the slow mode,

(32) $$\ddot{\Phi }+\left[\omega_{0}^{2}+\eta \left(t\right)\right]\Phi =\xi \left(t\right),$$

where η(t) and ξ(t) are not external noises but effective fluctuations generated by the internal SSM dynamics. In the notation introduced above,

(33) $$\eta \left(t\right)=2\mu \Omega^{2}\left[C_{2}\left(t\right)-{\bar{C}}_{2}\right],\;\xi \left(t\right)=-\mu \Omega^{2}S_{2}\left(t\right)-\bar{\ddot{\delta }}\left(t\right),$$

with

(34) $$C_{2}\left(t\right)\equiv \frac{1}{N}\sum_{i=1}^{N}\cos2\delta_{i},\;S_{2}\left(t\right)\equiv \frac{1}{N}\sum_{i=1}^{N}\sin2\delta_{i}.$$

In particular, η(t) represents a multiplicative random force, while ξ(t) describes the standard additive random force.

Instead of detailing the transformation from Equation (32) to an effective energy equation, we only summarize the result. Following the standard energy–angle reduction for noisy oscillators, one rewrites $\left(\Phi ,\dot{\Phi }\right)$ in terms of the energy–angle variables (E,χ), averages over the fast angle χ, and then coarse-grains over times longer than the bath correlation time. This yields an effective first-order stochastic dynamics for *E*,

(35) $$\dot{E}=aE+bE\eta +c\sqrt{E}\xi .$$

The multiplicative term bEη controls the finite-time log-normal regime, whereas the additive reinjection term $c\sqrt{E}\xi$ regularizes the process and allows stationary heavy tails. In the multiplicative-dominated regime, the last term drops in Equation (35). Applying Itô’s lemma to lnE gives

(36) $$\frac{d\ln E}{dt}=\left(a-b^{2}/2\right)+b\eta$$

Hence, lnE is Gaussian and the finite-time distribution of *E* is log-normal,

(37) $$P\left(E,t\right)=\frac{1}{E\sqrt{2\pi b^{2}t}}\exp\;\left[-\frac{{\left(\ln E-\ln E_{0}-\left(a-b^{2}/2\right)t\right)}^{2}}{2b^{2}t}\right].$$

This is the conservative-SSM counterpart of the simpler amplitude-modulation argument of Section 4.2: demodulation isolates the slowly varying collective envelope, while the chaotic internal sector acts as an effective multiplicative bath.

For the stationary statistics, one must retain the reinjection term $c\sqrt{E}\xi$. A simple one-variable Fokker–Planck description consistent with Equation (35) is

(38) $$\frac{\partial P}{\partial t}=-\frac{\partial }{\partial E}\left[aEP\right]+\frac{1}{2}\frac{\partial^{2}}{\partial E^{2}}\left[\left(b^{2}E^{2}+c^{2}E\right)P\right].$$

The large-*E* tail is controlled by the multiplicative diffusion term $b^{2}E^{2}$. If the reduced one-variable process admits a stationary state with vanishing probability current, then the large-*E* tail becomes algebraic,

(39) $$P_{\mathrm{st}}\left(E\right)\propto E^{-\alpha },\;\alpha =2-\frac{2a}{b^{2}}.$$

Thus, the same reduced description yields two complementary regimes: finite-time log-normal variability when multiplicative growth dominates, and a power-law stationary tail once reinjection is retained and a stationary Fokker–Planck balance is imposed. This reduced stochastic picture will be used again in Section 8 as the effective one-variable description of the collective mode extracted from the conservative many-body SSM.

### 4.4. A Nonequilibrium Viewpoint

The statistical behavior of the SSM is therefore neither purely equilibrium criticality nor purely stationary turbulence. It is better viewed as a nonequilibrium network of magnetic states whose coarse variables drift, synchronize, desynchronize, and occasionally reverse. In this picture, 1/f-like PSDs and heavy-tailed amplitudes are distinct projections of a single evolving collective process.

## 5. Unified Magnetic-Topological Model for Coronae, Flares, Winds, and Jets

The same collective variables that control the timing statistics can also classify the outflow and dissipation channels.

**Figure 7:**
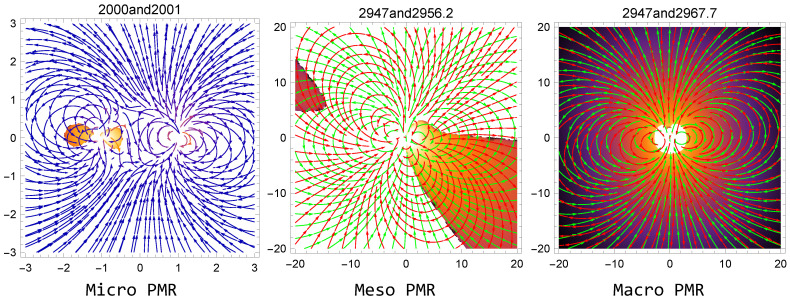
Magnetic-field comparison on the y=0 plane in the SSM simulation. The magnetic field at two nearby times is shown in different colors. Regions where the inner product of the two fields is negative are highlighted in orange or darker colors: the potential patch of magnetic reconnection and magnetic activity (PMR). The magnetic field is calculated as follows. We place *N* spins uniformly on a circle of radius 1 on the x-y plane centered at the origin. Assuming each spin is a magnetic moment, we superpose all the magnetic fields from the spins. **Left**: Micro PMR , the case of steady polarity. The reversed patch is small and local, but it persists at all times due to the persistent spin fluctuations. **Middle**: Meso PMR, the case of polarity excursion. The candidate zone of magnetic reconnection expands, yielding large active regions. **Right**: Macro PMR, the case of the full polarity flip. After a global polarity reversal, the field inner product is essentially negative everywhere, indicating a configuration in which magnetic energy can be efficiently converted into kinetic energy on large scales.

### 5.1. Closed Topology and Coronae

When the flux is predominantly closed, repeated local reconnection events heat the plasma without producing a strong organized outflow. Coronal luminosity will be expressed as

(40) $$L_{\mathrm{cor}}=A_{c}\left(1-f_{\mathrm{open}}\right)\left[Q_{0}+Q_{1}G\right],$$

where $A_{c}$ is a geometric conversion factor, $Q_{0}$ is a background dissipation level, and $Q_{1}G$ represents the reconnection-enhanced contribution. The factor $\left(1-f_{\mathrm{open}}\right)$ suppresses the corona when a large fraction of the flux is open. Equation (40) is therefore the simplest closure that increases with closed flux and with magnetic inhomogeneity.

Recent radiative PIC simulations have shown that magnetic reconnection in black-hole coronae can produce hard X-ray spectra through Comptonization by the mildly relativistic bulk motions of Compton-cooled plasmoids, especially in electron–ion plasmas where strong Compton cooling prevents the particles from remaining highly relativistic. This provides a kinetic-scale realization of the dissipative channel in the SSM: closed or sheared magnetic structures generated by partially synchronized macro-spins can form current sheets and power the corona through plasmoid-mediated reconnection [26,27].

**Figure 8:**
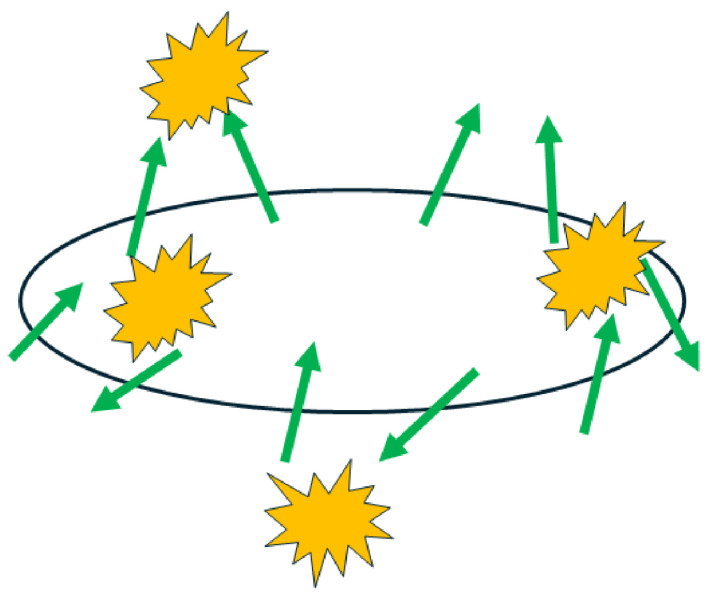
Schematic SSM picture of the corona generation. Coronal structures originate from the magnetically active region, starting from the magnetic reconnections (yellow area) above and below the region of the inner disk occupied by the coarse-grained spins (green). In the model, local and mesoscopic reconnection in this environment continuously heats the corona, whereas stronger large-scale ordering is required to form an axial jet channel.

Figure 8 sketches the corresponding geometry: the corona forms above and below the region in the inner disk where the magnetic domains or spins are located.

### 5.2. Partial Coherent Reversal and Flares

Flares are strongest when reconnection becomes coherent over a substantial fraction of the system without yet turning into a fully open axial structure. A simple phenomenological form of the flare power is

(41) $$P_{\mathrm{flare}}=A_{f}G\;\Theta (|M|-M_{c}),$$

where $A_{f}$ is a normalization constant, Θ is the Heaviside step function, and $M_{c}$ is the minimum global order required for large coherent events. The logic is that strong local gradients alone are not enough: the largest flares are expected when there is both substantial reconnection activity and enough large-scale organization to couple many sites together.

### 5.3. Partially Open Topology and Winds

Broad winds require open flux but not necessarily strong axial alignment. We therefore write the wind power as

(42) $$P_{\mathrm{wind}}=f_{\mathrm{open}}\left(1-C_{\mathrm{ax}}^{2}\right)\left[A_{w}S_{\mathrm{wind}}\left(\dot{m}\right)+B_{w}G\right],$$

where $\dot{m}$ is the dimensionless accretion rate, $S_{\mathrm{wind}}\left(\dot{m}\right)$ is a smooth source function for the quasi-steady wind background, and $B_{w}G$ measures the additional clumpy or intermittent contribution from magnetic restructuring. The prefactor $f_{\mathrm{open}}\left(1-C_{\mathrm{ax}}^{2}\right)$ encodes the intended geometry: wind power grows with open flux but is suppressed when the open flux becomes too strongly axis-aligned, in which case the system prefers a jet-like channel instead.

### 5.4. Open Axial Topology and Jets

Jets require both open flux and strong axial organization. A compact phenomenological closure for the jet power is

(43) $$P_{\mathrm{jet}}=A_{j}f_{\mathrm{open}}C_{\mathrm{ax}}^{2}M^{\alpha }E_{\mathrm{rot}}\left(\dot{m}\right)+B_{j}f_{\mathrm{open}}C_{\mathrm{ax}}^{2}\left|\frac{dM}{dt}\right|+C_{j}\Gamma_{\mathrm{rev},\mathrm{glob}},$$

where $E_{\mathrm{rot}}\left(\dot{m}\right)$ is an effective rotational energy supply function, α>0 controls how steeply steady jet power grows with large-scale order, and $\Gamma_{\mathrm{rev},\mathrm{glob}}$ is a phenomenological source term associated with global reversal or excursion. The first term represents the steady jet channel sustained by ordered open flux. The second term captures transient enhancement during rapid magnetic restructuring. The last term allows an additional burst-like contribution when global topology changes coherently. Equation (43) is therefore the simplest additive split between steady and impulsive jet channels.

## 6. Observational Connection I: Statistical Properties

We now compare the statistical diagnostics of the SSM with long-term X-ray variability data.

### 6.1. Main Agreement: The Demodulated Envelope Is the Relevant Variable

The clearest agreement concerns the demodulated signal rather than the raw one. In the SSM example of Figure 5, the detrended raw series is not pink, while the absolute value of the detrended series becomes pink and follows Taylor’s law. This is exactly the behavior expected if synchronization first generates an amplitude envelope and the low-frequency statistics are revealed only after a demodulation-like operation.

**Figure 9:**
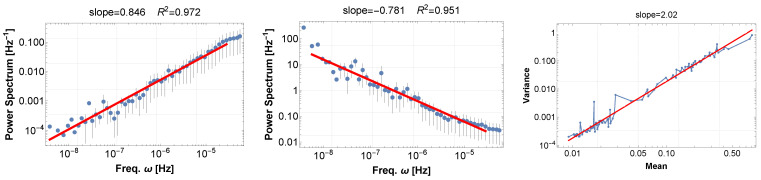
MAXI 16 yr diagnostics for GRS 1915+105, Galactic-Compact-Binary-BH, parallel to Figure 5. Left: PSD of the detrended light curve. Middle: PSD of the absolute value of the detrended light curve, which becomes pink. Right: Taylor’s law test. As in the SSM calculation, the demodulated envelope is the quantity that most clearly displays both pink noise and Taylor’s law behavior.

A similar pattern is seen observationally in at least part of the MAXI sample. Figure 9 shows that for GRS 1915+105 the detrended raw light curve is not pink, whereas the absolute value of the detrended series becomes pink and satisfies Taylor’s law. In this sense, the SSM captures a nontrivial qualitative feature of the data: the relevant long-memory structure may be hidden in the envelope and need not appear directly in the signed fluctuation itself.

### 6.2. Main Mismatch: SSM Appears to Overproduce This Behavior

The problem is that the present SSM tends to produce this transformation too broadly. Even for parameter sets where the raw detrended SSM series is not pink, taking the absolute value often makes it pink and Taylor-like. In the model, hidden-envelope statistics are therefore rather generic.

The MAXI data are more selective. Empirically, sources that are already close to pink in the original light curve often remain pink and Taylor-like after the absolute-value operation. However, for many sources that are not pink to begin with, taking the absolute value does not produce pink noise or Taylor’s law. Thus the observational class “non-pink raw → pink absolute value” certainly exists, but it is not nearly as universal as in the present SSM.

### 6.3. Interpretation

This mismatch suggests that synchronization and nonlinear demodulation are important but not sufficient. The current SSM captures the mechanism by which envelope extraction can reveal hidden long-timescale organization, but it does not yet include enough of the additional physics that determines whether such organization survives in the observed X-ray flux.

The most likely missing ingredients are straightforward. The observed flux is not the order variable itself but a radiatively filtered observable. Real sources also contain additive contamination from other variability channels, state mixing, and finite observational windows. Any of these can suppress the pink/Taylor signature even if the underlying magnetic dynamics contains an envelope hierarchy. In this sense, the present SSM is best viewed as describing one important variability component rather than the full observational transfer problem.

These issues lead us to go beyond static diagnostics and investigate the detailed synchronization history across the entire observation time series.

### 6.4. Time-Dependent Variation of the PSD Slope: Comparison of Figure 6 and Figure 10

**Figure 10:**
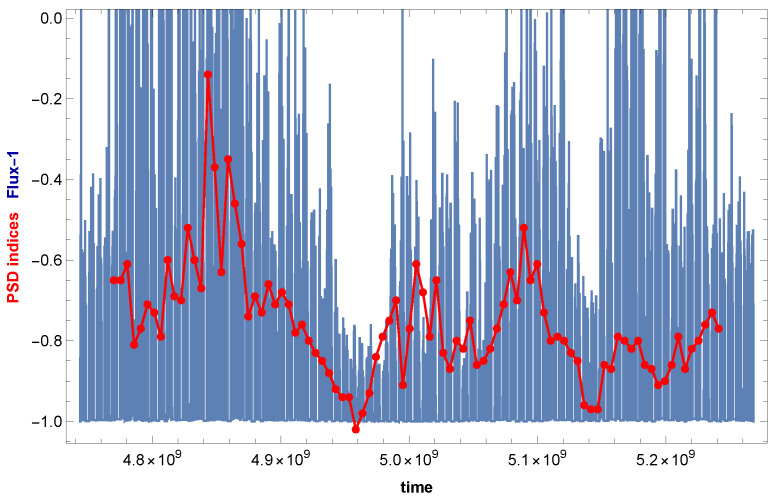
MAXI 16 yr running-index analysis for Cyg X-1, parallel to Figure 6. Shown are the normalized X-ray flux shifted downward by 1 (blue) and the running PSD indices (red) obtained using the same method as in Figure 6. The PSD slope tends to be steeper at lower X-ray intensity and flatter, closer to white noise, at higher intensity. This suggests that the degree of long-timescale synchronization changes across the observed long-term variability pattern. This may not be a single transient outburst q-cycle, but rather with state wandering, failed transitions, and secular changes in the accretion or magnetic-correlation structure.

A further important point is the time dependence of the PSD slope within a single data set. The comparison between the SSM result in Figure 6 and the MAXI result in Figure 10 suggests that the low-frequency PSD index is not a fixed property of a source but varies in time together with the degree of large-scale ordering.

Figure 6 suggests that, within the SSM, the running PSD index changes systematically along the same time series as the collective spin variable. In particular, during polarity reversals or large excursion-like episodes, the PSD slope tends to become white (the slope shallower than pink), indicating that strong global rearrangement weakens the frequency crowding and partial synchronization that otherwise support the low-frequency continuum. Conversely, during more ordered epochs, the PSD is more likely to approach a pink spectrum. Thus, the PSD index and the degree of collective spin ordering are dynamically linked within one realization.

Figure 10 suggests an observational counterpart of the same idea. In the MAXI light curve, the running PSD index also changes within a single source over time, rather than remaining constant. This indicates that the observed spectrum is evolving together with the source state. Although the observational quantity is not the spin order parameter itself, the qualitative similarity to Figure 6 is suggestive: in both the model and the data, the spectral index moves as the internal state of the system changes.

The state dependence of the long-term PSD should not be regarded as an artifact of the moving-window analysis. Sugimoto [28] has already shown, using MAXI data, that the long-term PSD differs between the hard and soft states down to ≈10^−7^ Hz, with the states classified independently by the hardness ratio. Thus, the dependence of the PSD morphology on the accretion state is an observationally motivated physical fact.

Figure 10 extends this idea by showing the moving-window PSD slope as a time-dependent diagnostic. We do not identify each point of this time series with a unique spectral state. Instead, the moving-window slope should be regarded as an effective finite-band measure of non-stationary broad-band variability. It may reflect both relatively rapid changes associated with state transitions or failed transitions and slower secular changes in the magnetic correlation structure or state occupation fraction.

In the SSM, the PSD slope is expected to depend on the synchronization state of the macro-spins, the distribution of magnetic correlation times, and the relative weights of coherent and incoherent magnetic activity. Therefore, a temporal change in the effective PSD slope is a natural consequence of a time-dependent magnetic-order state.

This dynamical viewpoint motivates the next section. If the PSD slope varies together with the degree of ordering in both the SSM and the observed light curve, then the statistical properties and the large-scale state evolution should be understood within a common framework rather than as separate phenomena. In the following section, we therefore examine this evolving state structure more explicitly.

## 7. Observational Connection II: Cycles

### 7.1. Minimal Three-Level Phenomenological Variables for the q-Diagram

The micro/meso/macro PMR labels in Figure 7 should be understood as a geometric illustration of the same multilevel viewpoint later encoded phenomenologically by the reduced variables m(t),s(t),M(t), rather than as a one-to-one definition of those variables.

**Figure 11:**
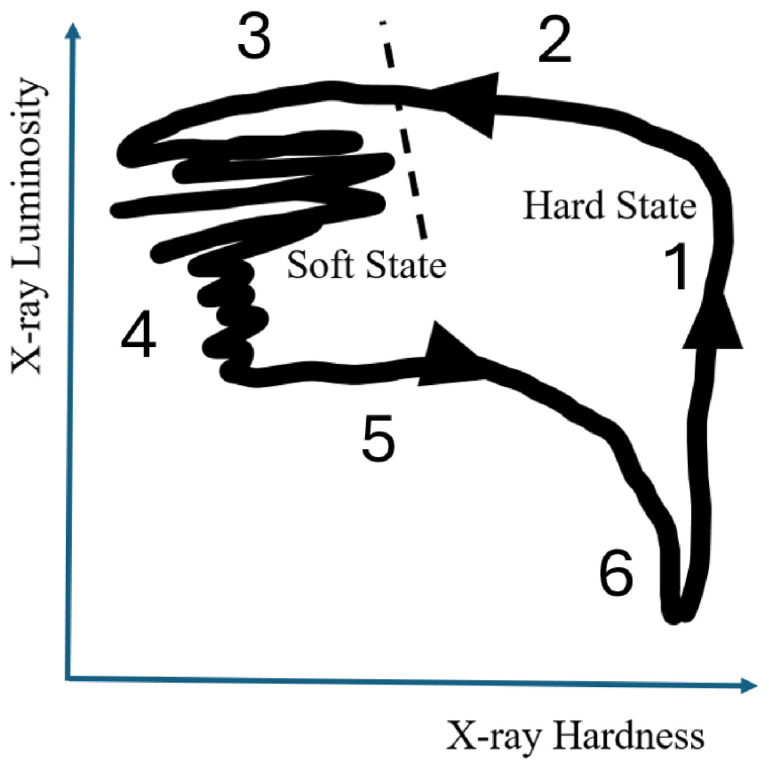
Canonical hardness–intensity diagram (q-diagram) of black-hole X-ray binaries. The SSM interpretation assigns the hard branch (1) to a noisy but globally organized magnetic state, through the intermediate state (2), the jet-line region (3) to rapid large-scale magnetic restructuring with strong transient ejecta, and the soft branch (4) to a thermally stable but less dipolar magnetic configuration with suppressed steady jet power. Through quiet transient states (5, 6), the system returns to the hard state (1).

Figure 11 summarizes the phenomenological state cycle, or q-diagram, to be interpreted. The SSM reading is that hard states retain comparatively strong macro-order and open axial flux, the jet-line region corresponds to rapid restructuring with strong transient ejecta, and soft states suppress the steady jet because the global dipole-like order is weaker even though local magnetic activity remains present.

To formalize this picture, we introduce four reduced variables,

m(t)≥0: micro-scale fluctuation intensity: level-1 = micro,
s(t)∈[0,1]: meso-scale synchronization or clustering: level-2 = meso,
M(t)∈[−1,1]: signed macro-scale dipolar order parameter: level-3 = macro,
H(t)≥0: history variable storing helicity, free magnetic energy, or topology memory.

The purpose of this set is not uniqueness, but economy: *m* captures fast variability, *s* captures intermediate coherence, *M* captures large-scale polarity and order, and *H* introduces hysteresis.

### 7.2. Minimal Dynamical System

The following equations are not derived from first-principles GRMHD. They are a reduced phenomenological model designed to capture the observed state sequence, hysteresis, and the distinction between steady and transient jet channels.

Micro-scale activity

(44) $$\frac{dm}{dt}=\epsilon_{0}+\epsilon_{1}{\left(\frac{dM}{dt}\right)}^{2}+\epsilon_{2}s-\gamma_{m}m.$$

Here, $\epsilon_{0}$ is a baseline fluctuation source, $\epsilon_{1}{\left(dM/dt\right)}^{2}$ expresses additional agitation during rapid macro-scale restructuring, $\epsilon_{2}s$ allows meso-scale synchronization to feed smaller-scale variability, and $\gamma_{m}$ is a decay rate.

Meso-scale synchronization

(45) $$\frac{ds}{dt}=\alpha_{s}\;m\left(1-s\right)+\beta_{s}\left|M\right|\left(1-s\right)-\chi_{s}s-\eta_{s}{\left(\frac{dM}{dt}\right)}^{2}s.$$

The two growth terms encode the idea that both micro-scale stirring and existing macro-order can seed meso-scale clustering, while the factors (1−s) saturate the growth as s→1. The term $\chi_{s}s$ is a linear loss term, and $\eta_{s}{\left(dM/dt\right)}^{2}s$ suppresses meso-scale coherence during violent global rearrangement.

Macro-scale dipolar order

(46) $$\frac{dM}{dt}=a\left(H,m\right)\;M-bM^{3}+c\;s-d\;\Xi \;M+\xi_{M}\left(t\right),$$

with

(47) $$a\left(H,m\right)=a_{0}+a_{H}H-a_{m}m.$$

Here, $a_{0}$ is the baseline linear growth coefficient, $a_{H}H$ lets stored history stabilize large-scale order, and $a_{m}m$ lets strong micro-turbulence weaken it. The cubic term $-bM^{3}$ is the standard Landau saturation that prevents unbounded growth. The term cs says that meso-scale synchronization can seed macro-order. The factor dΞM allows accretion geometry or thermodynamic state, summarized by Ξ, to suppress or reshape the large-scale field. Finally, $\xi_{M}\left(t\right)$ is a stochastic forcing term.

History variable and hysteresis

(48) $$\frac{dH}{dt}=\mu_{H}\left|M\right|s-\nu_{H}{\left(\frac{dM}{dt}\right)}^{2}H-\gamma_{H}H.$$

The source term $\mu_{H}\left|M\right|s$ states that helicity or free magnetic energy is stored when meso- and macro-order coexist. The term $\nu_{H}{\left(dM/dt\right)}^{2}H$ releases that stored energy during rapid reorganization, while $\gamma_{H}H$ describes slow relaxation. This is the minimal mechanism by which hysteresis enters the q-diagram interpretation.

The structure of Equations (44)–(48) is that of a nonlinear feedback oscillator in magnetic-state space. Micro-scale activity m promotes meso-scale clustering s, which in turn seeds macro-scale dipolar order *M*. When *s* and *M* coexist, the history variable *H*, representing helicity or free magnetic energy, is slowly accumulated. However, the same micro-scale activity also suppresses the linear growth rate of *M* through a(H,m) (Equation (47)), and rapid changes of *M* destroy *s* and release *H* through the ${\dot{M}}^{2}$ terms. Thus the system contains the ingredients of a relaxation cycle: slow magnetic ordering and energy storage, followed by rapid restructuring and release, and then recovery. In this sense the q-diagram need not be imposed externally, but can be interpreted as a low-dimensional magnetic-state cycle, analogous in spirit to low-dimensional nonlinear models such as the Lorenz model for fluid convection or predator–prey cycles in population dynamics.

### 7.3. Directionality of the q-Diagram

The observed q-diagram is directional: the hard-to-soft and soft-to-hard transitions do not follow the same path. This hysteresis is naturally incorporated in the SSM because the magnetic order parameter is history-dependent. Magnetic flux accumulation, synchronization of local dynamo domains, reconnection, and magnetic energy release are dissipative nonequilibrium processes. Therefore, the destruction of an ordered magnetic state during the hard-to-soft transition is not simply the reverse of the gradual rebuilding of magnetic order during the soft-to-hard transition. In this view, transient/discrete jets are preferentially associated with the hard-to-soft transition because this is the phase in which a previously accumulated coherent magnetic structure becomes unstable and releases energy impulsively. The return to the hard state corresponds instead to the slower reformation of the ordered magnetic configuration and does not necessarily produce an equally strong discrete ejection.

In the SSM, the hard state is interpreted as a long-lived magnetically ordered branch of the disk–corona–jet system. During this phase, local dynamo domains gradually develop a coherent large-scale magnetic order, which supports a compact steady jet and strong broad-band variability. The hard-to-soft transition is then associated with rapid destruction, reversal, or large-scale reconnection of this ordered magnetic configuration, producing transient/discrete ejecta. The subsequent soft state corresponds to a magnetically disordered or chaotic phase, in which the global order parameter is strongly reduced although local magnetic activity may persist. A new ordered configuration, possibly with the opposite polarity, can then be rebuilt, returning the system to the hard branch. Thus, the SSM naturally produces long intervals of nearly fixed magnetic polarity separated by short reversal or reconnection events. In this sense, the hard state may be regarded as the natural magnetic baseline, or attractor branch, of the SSM cycle, rather than merely as one spectral state among others.

### 7.4. State-by-State Interpretation of the q-Diagram

The resulting interpretation is summarized in Table 1. The hard state (1) is noisy but globally ordered; the jet-line region (3) is a restructuring zone with large |dM/dt|; the soft state (4) is thermally stable but magnetically less dipolar; and the decay branch (5) is not the mirror image of the rise because the history variable *H* produces hysteresis.

## 8. Quantitative Tests and Observational Falsifiability of the SSM Scenario

In this section, we present the main order-of-magnitude checks and falsifiable predictions of the synchronized spin model (SSM) for cosmic magnetic activity. The basic viewpoint is that the dominant energy reservoir is the gravitational potential energy released by accretion, while magnetic fields store, redistribute, and intermittently release a fraction of this energy through reconnection events on various scales. Thus, the SSM yields quantitative expectations for the energetics of discrete jet knots, the hierarchical relation among jet–flare–corona phenomena, alternating knot amplitudes, magnetic polarity relics, and the response of AGN luminosity to rapid changes in the accretion state [9,31,32].

### 8.1. Energetic Consistency from Discrete Jets to the Corona

(i) knot energy budget

As a fiducial upper-envelope estimate, let us denote by $E_{B,\max}$ the magnetic energy that can participate in one major knot-launching episode associated with a large-scale reversal or excursion. The important point is that this should not be interpreted as the instantaneous magnetic energy of a single compact blob of size ∼100 *r_g_*. Observational and theoretical studies of AGN jets increasingly indicate that acceleration and collimation proceed over an extended acceleration–collimation zone (ACZ), often reaching ≲10^5^–10^6^ *r_g_* from the black hole [33,34]. We therefore adopt a more realistic effective scale,

(49) $$B∼300\;G,\;R_{\mathrm{eff}}∼2000\;r_{g},$$

for the inner magnetically active part of the ACZ that may participate coherently in a single major release episode. This choice is still conservative in the sense that $R_{\mathrm{eff}}$ is far smaller than the full observed ACZ extent.

With this interpretation, the available magnetic energy scales as

(50) $$E_{B,\max}∼\frac{B^{2}}{8\pi }\;\frac{4\pi }{3}R_{\mathrm{eff}}^{3}\propto B^{2}R_{\mathrm{eff}}^{3},$$

and the above setting yields the order estimate,

(51) $$E_{B,\max}∼\mathrm{few}\times 10^{53}-10^{54}\;\mathrm{erg},$$

which is the scale relevant for the largest AGN knot-launching episodes discussed below. In this reading, $E_{B,\max}$ represents the effective magnetic reservoir of the inner ACZ participating in one major event, rather than the magnetic content of a single compact plasmon.

Introducing an efficiency $\eta_{k}$ for the conversion of magnetic energy into the bulk energy of a discrete knot, the ejecta energy is

(52) $$E_{\mathrm{knot}}≃\eta_{\mathrm{rec}}\eta_{\mathrm{bulk}}E_{B}\equiv \eta_{k}E_{B},\;0<\eta_{k}<1.$$

The remainder is naturally expected to be partitioned into thermal energy, non-thermal particles, radiation, and residual Poynting flux [31,35].

If the knot is ejected with Lorentz factor Γ, its bulk kinetic energy is

(53) $$E_{\mathrm{knot}}=\left(\Gamma -1\right)M_{\mathrm{knot}}c^{2}.$$

Using the fiducial upper-envelope value above, one obtains

(54) $$M_{\mathrm{knot}}≃1.0\;\eta_{k}\left(\frac{E_{B}}{8\times 10^{53}\;\mathrm{erg}}\right){\left(\frac{\Gamma -1}{0.45}\right)}^{-1}M_{⊙},$$

where Γ≃1.45 corresponds to β≃0.7. Thus, for a mildly relativistic plasmon, a knot mass of order $M_{\mathrm{knot}}∼M_{⊙}$ is not inconsistent with the fiducial upper-envelope estimate. For faster knots, the implied mass decreases rapidly, $M_{\mathrm{knot}}∼0.1\;\eta_{k}\;M_{⊙}\;\left(\Gamma ≃5.5,\;\beta ≃0.98\right),$ and $M_{\mathrm{knot}}∼0.05\;\eta_{k}\;M_{⊙}\;\left(\Gamma ≃10,\;\beta ≃0.995\right).$ Therefore, the SSM does not require a unique knot mass.

(ii) hierarchical energy-per-decade

A useful extension of this argument is obtained if a suitably defined catalog of reconnection-driven events obeys an approximate occurrence distribution

(55) $$\frac{dN}{dE}=AE^{-\alpha },\;\alpha ≃2,$$

as often discussed for flare-like avalanches and SOC-like systems [11,36]. Here, the key point is interpretive: this form is not assumed to describe the full continuous flux distribution of AGN/XRB light curves, which is often closer to log-normal in the multiplicative-variability picture [7]. Instead, it is used as an effective event-catalog model for the subset of reconnection-driven bursts that can be identified as discrete flares, shots, or ejecta. In solar and stellar flare systems, this approximation is often directly useful, while in black-hole systems, it should be regarded as an illustrative hypothesis whose validity depends on event definition and source state [16,37].

The energy released *W* per logarithmic interval is then

(56) $$\frac{dW}{d\ln E}=E\frac{dN}{d\ln E}=E^{2}\frac{dN}{dE}=AE^{2-\alpha }.$$

For α=2, this becomes

(57) $$\frac{dW}{d\ln E}=A=\mathrm{const}.,$$

so that each decade or octave in event energy carries approximately the same total released energy. In the SSM context, this provides a convenient energetic bridge between the largest identified events (discrete jet ejections), intermediate events (large flares or clumpy wind ejecta), and the smallest identified events (unresolved reconnection bursts contributing to coronal heating).

Here, $E_{\max}∼10^{54}\;\mathrm{erg}$ should be understood as the upper end of the effective event hierarchy associated with major ACZ-scale magnetic episodes, not as the energy of a single elementary reconnection site. If $E_{\max}∼10^{54}$ erg and $E_{\min}∼10^{42}$ erg, the hierarchy spans $\log_{10}\;\left(E_{\max}/E_{\min}\right)∼12$ decades, and therefore the average power deposited per decade is roughly

(58) $$L_{\mathrm{per}\;\mathrm{decade}}∼\frac{L_{\mathrm{mag}}}{\log_{10}\left(E_{\max}/E_{\min}\right)}∼\frac{L_{\mathrm{mag}}}{12},$$

where $L_{\mathrm{mag}}$ is the total luminosity associated with magnetic activity. This means that, once the magnetic budget is sufficient to power rare giant knot-launching events, it is energetically plausible that lower-energy bands of the same hierarchy can sustain persistent flaring and coronal heating.

A convenient way to interpret the power scale used below is to convert the largest knot- launching event into a time-averaged magnetic power per logarithmic energy interval. If the upper-end episode has characteristic energy $E_{\max}∼10^{54}\;\mathrm{erg}$ spanning about one decade, and occurs once per $\Delta t_{\max}∼300\;\mathrm{yr}∼10^{10}\;s$, then

(59) $$L_{\mathrm{per}\;\mathrm{decade}}∼\frac{E_{\max}}{\Delta t_{\max}}∼10^{44}\;\mathrm{erg}\;s^{-1},$$

suggesting comparable power per logarithmic interval across the jet, wind, and coronal bands, if such a hierarchy is realized.

(iii) corona/wind consequences

A simple consistency check of the coronal temperature $T_{\mathrm{cor}}$, coronal luminosity $L_{\mathrm{cor}}$, and coronal energy $U_{\mathrm{cor}}$ follows from

(60) $$U_{\mathrm{cor}}∼3n_{\mathrm{cor}}V_{\mathrm{cor}}k_{B}T_{\mathrm{cor}},$$

together with

(61) $$U_{\mathrm{cor}}∼L_{\mathrm{cor}}t_{\mathrm{res}},$$

where $n_{\mathrm{cor}}$, $V_{\mathrm{cor}}$, and $t_{\mathrm{res}}$ are the coronal density, volume, and effective energy residence time. One then finds

(62) $$T_{\mathrm{cor}}∼\frac{L_{\mathrm{cor}}t_{\mathrm{res}}}{3n_{\mathrm{cor}}V_{\mathrm{cor}}k_{B}}.$$

If, owing to the approximately equal-energy-per-decade property, the coronal heating band receives a fraction $\epsilon_{\mathrm{cor}}$ of the total magnetic power, $L_{\mathrm{cor}}∼\epsilon_{\mathrm{cor}}L_{\mathrm{mag}}$, then

(63) $$T_{\mathrm{cor}}∼7.2\times 10^{8}\;K\left(\frac{\epsilon_{\mathrm{cor}}L_{\mathrm{mag}}}{10^{44}\;\mathrm{erg}\;s^{-1}}\right)\left(\frac{t_{\mathrm{res}}}{10^{4}\;s}\right){\left(\frac{n_{\mathrm{cor}}}{10^{9}\;{\mathrm{cm}}^{-3}}\right)}^{-1}{\left(\frac{V_{\mathrm{cor}}}{10^{46}\;{\mathrm{cm}}^{3}}\right)}^{-1}.$$

This is deliberately a one-zone upper-envelope estimate, but it shows that X-ray-coronal temperatures of order $10^{8}$–$10^{9}$ K are not energetically problematic if only a modest fraction of the SSM magnetic budget is thermalized in a compact corona. Here, $t_{\mathrm{res}}∼10^{4}$ s is adopted as a fiducial residence time appropriate for a compact AGN corona, corresponding to an order-of-magnitude light-crossing/escape/dissipation timescale of a region of several to several tens of $r_{g}$ around a $10^{8}\;M_{⊙}$ black hole.

A similar order-of-magnitude estimate may be made for the wind velocity if a fraction $\epsilon_{w}$ of the magnetic power is deposited into clumpy wind ejecta rather than into the corona or the jet. Writing the wind kinetic power as

(64) $$L_{w}∼\frac{1}{2}{\dot{M}}_{w}v_{w}^{2},$$

one obtains

(65) $$v_{w}∼{\left(\frac{2L_{w}}{{\dot{M}}_{w}}\right)}^{1/2}≃4.0\times 10^{8}{\left(\frac{L_{w}}{10^{43}\;\mathrm{erg}\;s^{-1}}\right)}^{1/2}{\left(\frac{{\dot{M}}_{w}}{1\;M_{⊙}\;{\mathrm{yr}}^{-1}}\right)}^{-1/2}\mathrm{cm}\;s^{-1}.$$

Thus, even for a moderate mass-loading rate, reconnection-powered wind ejecta can naturally reach velocities of order $10^{3}$–$10^{4}\;\mathrm{km}\;s^{-1}$. For smaller ${\dot{M}}_{w}$ or more concentrated energy deposition, the same estimate extends into the ultra-fast outflow regime, $v_{w}∼0.1c$, consistent with observed AGN ultra-fast outflows and with the recent XRISM evidence that at least some quasar winds are highly structured rather than smooth and continuous [19,38]. This suggests that at least part of the observed AGN wind phenomenology may be better viewed not as a perfectly continuous flow, but as a hierarchy of discrete, bullet-like ejecta driven by multiscale magnetic reconnection.

In the SSM picture, such wind bullets occupy an intermediate part of the same reconnection hierarchy that connects the largest jet-launching events to the smallest coronal-heating events. By contrast, if the relevant event-energy distribution is closer to log-normal,

(66) $$p\left(E\right)=\frac{1}{E\sigma \sqrt{2\pi }}\exp\;\left[-\frac{{\left(\ln E-\mu \right)}^{2}}{2\sigma^{2}}\right],$$

then the energy released per logarithmic interval becomes

(67) $$\frac{dW}{d\ln E}\propto E^{2}p\left(E\right),$$

which peaks around a characteristic scale $E_{*}=\exp\left(\mu +\sigma^{2}\right)$ rather than remaining flat across decades. Hence, the equal-energy-per-decade argument is specific to the effective power-law event-catalog approximation and should not be confused with the log-normal description of the continuous multiplicative variability.

### 8.2. Alternating Knot Amplitudes and the Doubled Cycle

A distinctive SSM prediction is that if a slowly varying large-scale background field $B_{g}$ coexists with a polarity-reversing dynamo component $B_{d}$, then successive knot-launching events need not be energetically identical. In the simplest picture, the reconnecting field alternates between constructive and destructive combinations,

(68) $$B_{\mathrm{rec},\pm }∼B_{d}\pm B_{g},$$

so that the large-to-small knot energy ratio is estimated as

(69) $$\frac{E_{\mathrm{large}}}{E_{\mathrm{small}}}={\left(\frac{B_{d}+B_{g}}{B_{d}-B_{g}}\right)}^{2}.$$

For example,

(70) $$\begin{array}{cc} \frac{B_{g}}{B_{d}}=0.1\; & \Rightarrow \;\frac{E_{\mathrm{large}}}{E_{\mathrm{small}}}={\left(\frac{1.1}{0.9}\right)}^{2}≃1.49, \end{array}$$

(71) $$\begin{array}{cc} \frac{B_{g}}{B_{d}}=0.2\; & \Rightarrow \;\frac{E_{\mathrm{large}}}{E_{\mathrm{small}}}={\left(\frac{1.2}{0.8}\right)}^{2}=2.25. \end{array}$$

Thus, even a background field at the 10–20% level of the reversing field can generate a robust factor-of-1.5–2 alternating modulation in successive knot amplitudes. Observationally, one may search for this by fitting the knot flux sequence $F_{n}$ with

(72) $$F_{n}=\bar{F}\left[1+\epsilon \cos(\pi n+\phi )\right],$$

or equivalently by testing whether neighboring ratios $R_{n}\equiv F_{n+1}/F_{n}$ exhibit a statistically significant even–odd asymmetry. In this picture, the knot spacing traces the fundamental reversal cadence, while the brightness alternation traces the doubled cycle.

A complementary timing estimate comes from the deprojected knot spacing Δr and the intrinsic knot speed βc:

(73) $$T_{\mathrm{ej}}∼\frac{\Delta r}{\beta c}.$$

For the quasi-periodic knot train in PKS 0637−752, the observed projected separation is of order $\Delta r_{\mathrm{proj}}∼7.6\;\mathrm{kpc}$ [39]. After deprojection, the inferred modulation timescale is of order $10^{3}$–$10^{5}$ yr, depending on the jet speed and geometry [39]. Within the SSM, this is interpreted as an estimate of the large-scale magnetic reversal timescale. The same logic can be applied to systems of very different masses by using the dimensionless ratio

(74) $$\frac{T_{\mathrm{ej}}}{t_{g}},\;t_{g}\equiv \frac{GM_{•}}{c^{3}},$$

which provides a direct route to testing whether protostellar jets, microquasars, and AGN jets lie on a common hierarchy when measured in gravitational time units.

### 8.3. Magnetic Polarity Relics in the Circumnuclear Medium

If a sequence of SSM reversals repeatedly injects magnetic flux of alternating polarity into the surrounding medium, the resulting field geometry may leave a spatially stratified relic pattern, provided that magnetic advection dominates over turbulent diffusion on the corresponding scale. The characteristic width of one polarity domain is

(75) $$\lambda_{\mathrm{rev}}∼v_{\mathrm{adv}}T_{\mathrm{rev}},$$

where $v_{\mathrm{adv}}$ is an effective transport speed and $T_{\mathrm{rev}}$ is the reversal period. Adopting representative nuclear values motivated by [40],

(76) $$\begin{array}{cc} v_{\mathrm{adv}} & ∼100\;\mathrm{km}\;s^{-1}, \end{array}$$

(77) $$\begin{array}{cc} T_{\mathrm{rev}} & ∼10^{4}\;\mathrm{yr}, \end{array}$$

one finds

(78) $$\lambda_{\mathrm{rev}}∼\left(10^{7}\;\mathrm{cm}\;s^{-1}\right)\left(3.15\times 10^{11}\;s\right)∼3\times 10^{18}\;\mathrm{cm}∼1\;\mathrm{pc}.$$

For $T_{\mathrm{rev}}∼10^{5}$ yr, this becomes

(79) $$\lambda_{\mathrm{rev}}∼10\;\mathrm{pc},$$

and somewhat larger values follow if $v_{\mathrm{adv}}∼300\;\mathrm{km}\;s^{-1}$. Therefore, the most promising search region is not the entire galactic disk, but the central parsec-to-tens-of-parsecs environment, where alternating sign changes in polarization or Faraday rotation may survive as a fossil record of nuclear magnetic reversals. Mapping of magnetic-field structure in the Galactic environment already shows that coherent field reversals can be observationally reconstructed [41].

The competing process is turbulent diffusion, with a characteristic timescale

(80) $$t_{\mathrm{diff}}∼\frac{L^{2}}{\eta_{\mathrm{turb}}}.$$

Using a fiducial turbulent diffusivity consistent with [42],

(81) $$L∼100\;\mathrm{pc},\;\eta_{\mathrm{turb}}∼10^{26}\;{\mathrm{cm}}^{2}\;s^{-1},$$

one obtains

(82) $$t_{\mathrm{diff}}∼\frac{{\left(3\times 10^{20}\;\mathrm{cm}\right)}^{2}}{10^{26}\;{\mathrm{cm}}^{2}\;s^{-1}}∼10^{15}\;s∼3\times 10^{7}\;\mathrm{yr}.$$

Hence, parsec-scale to sub-kiloparsec-scale polarity relics are, at least in principle, long-lived enough to be observable, whereas clean signatures over full spiral-arm scales are expected to be more easily erased by galactic shear and turbulence.

The tomography of the Sagittarius arm [41] shows that pc-scale coherent magnetic patches are observationally plausible, with smooth field structure on scales below ∼10pc. However, those clouds lie at Galactocentric radii of ∼6–7kpc, so they should be regarded as an existence proof of such coherence rather than as a direct test of the nuclear fossil-polarity scenario proposed here.

### 8.4. Rapid AGN Fading and the Immediate Energy Source

An interesting observational test is provided by AGN that fade dramatically on decadal timescales. The quasar SDSS J021801.90-003657.7, for example, showed a decline from SDSS to HSC photometry by factors of order 10–20 in the optical over roughly two decades [32,43]. If the instantaneous radiative output $L_{\mathrm{rad}}$ is controlled primarily by accretion power, one expects

(83) $$L_{\mathrm{rad}}≃\eta \dot{M}c^{2},$$

with radiative efficiency η∼0.1 in a standard disk. A luminosity decline by a factor *f* then corresponds, at the crudest level, to

(84) $${\dot{M}}_{f}≃\frac{{\dot{M}}_{i}}{f}.$$

This argument is not unique to SSM: even in more standard accretion-disk pictures, a reduced mass supply to the inner disk naturally leads to a long-lived decline of activity. The point of the present example is therefore narrower. Such fading events support the general premise that the immediate power source of observed AGN activity is the gravitational energy released by accretion, not an order-unity change in the black-hole spin reservoir on decadal timescales.

What is more specific to the SSM picture is how the activity channels are reorganized during that decline. Since SSM links coronae, flares, winds, and jets to a common evolving magnetic state, the more discriminating test is whether these channels vary independently or in a correlated manner during a fading event. In this sense, the observational target is not the luminosity drop alone, but the pattern of channel rearrangement: whether coronal activity, flare occurrence, wind signatures, and jet/non-jet behavior are reorganized coherently as the system moves through magnetic state space.

### 8.5. Additional Falsifiable Diagnostics

Beyond the tests discussed above, the SSM yields at least three further semi-quantitative diagnostics.

First, the SSM predicts a symmetric bipolar ejection in both directions simultaneously associated with a polarity flip event. Thus, it predicts that the initial energies of the two opposite knots should satisfy

(85) $$E_{+}≃E_{-},$$

up to projection and Doppler effects. Hence, after correcting for beaming as far as possible, the intrinsic energetics of the two sides should be more symmetric than in models where the observed knot structure is generated mainly by environmental shocks. Twin-jet systems such as NGC 1052 provide useful laboratories for this check [44].

Second, if large flares and knot ejections are different manifestations of the same reversal-driven reconnection event, the appearance of a new knot should follow a major flare after a propagation delay of order

(86) $$\Delta t_{\mathrm{flare}\to \mathrm{knot}}∼\frac{r_{\tau ∼1}}{\beta c},$$

where $r_{\tau ∼1}$ is the photospheric or radio-core distance at which the new knot becomes visible. Simultaneous multi-wavelength monitoring and VLBI imaging can test this directly.

Third, the jet width and collimation profile carry an imprint of how magnetically organized the outflow remains after launch. The observed collimation properties of systems such as 3C 273 [45] provide a geometric constraint complementary to energetics: if the knot train is truly a sequence of compact magnetized plasmons rather than a quasi-continuous fluid, then long-distance collimation becomes less surprising, because each knot transports its own internal magnetic and kinetic structure.

Taken together, these tests show that the SSM is falsifiable in several independent ways. The model is supported if one finds: (i) knot-launching energetics compatible with a reconnection-powered magnetic reservoir; (ii) a roughly equal-energy hierarchy across jet, flare, and coronal bands when analyzed per logarithmic interval; (iii) an even–odd modulation of successive knot amplitudes consistent with a doubled cycle; (iv) parsec-scale relic polarity domains around nuclei; and (v) rapid AGN fading events whose energetics track accretion-state changes rather than changes in black-hole spin energy. Conversely, the model would be disfavored if high-quality data were to show that knot sequences are purely stochastic with no doubled-cycle component, that no bipolar energetic symmetry exists even after deprojection, or that the full AGN power output remains unchanged despite order-of-magnitude changes in the inferred inner accretion rate.

## 9. Discussion

We briefly outline the SSM’s niche within existing theories in this field.

1. What SSM adds
  SSM is not a replacement for GRMHD, radiative transfer, BZ/BP launching, or reconnection microphysics. Its role is narrower: it provides a mesoscopic state language that links variability, partial coherence, magnetic topology, and outflow morphology within one reduced variable set.
2. Relation to existing frameworks
  Propagating-fluctuation and SOC-like accretion-cascade models explain broad-band timing by fluctuations or avalanches in the accretion flow. Reconnection or plasmoid models explain impulsive magnetic dissipation. BZ/BP frameworks explain launching once an ordered open magnetic geometry exists. The niche of the SSM is not to replace any of these components, but to connect them through a mesoscopic magnetic state. In this view, accretion controls the slow energy supply and background disk structure; magnetic synchronization controls the coherence and topology of the stored field; and reconnection provides the rapid conversion process that partitions the stored magnetic energy into coronae, flares, winds, and steady or transient jets. This is why the SSM is formulated as a state theory rather than as a microscopic reconnection model or a source-by-source GRMHD simulation.
3. Limitations
  The mapping from GRMHD fields to macro-spin domains remains phenomenological, the effective coefficients are not derived from first principles, and radiative and thermal physics are compressed into source functions. The present framework should therefore be regarded as a reduced-state language rather than as a source-by-source predictive simulation model.
4. A possible validation route for the SSM
  The SSM is intentionally simple and phenomenological, and it is not intended as a direct derivation from GRMHD or as a source-by-source predictive model. Its value lies instead in providing a compact description of collective magnetic behavior in rotating conducting fluids. From this perspective, a possible route to validation is to ask whether similar multilevel magnetic activity can be identified across a wider range of cosmic systems, including laboratory dynamos [46,47,48].
  The SSM suggests similarities between apparently different systems. For example, the q-diagram in XRBs and the solar cycle may be viewed as different realizations of synchronize–desynchronize cycles, and the shift of solar-flare PSD indices from pink toward whiter spectra near solar maximum suggests the same link between spectral slope and global magnetic reorganization. At the laboratory scale, the VKS (von Kármán sodium) experiment provides an instructive analog: a turbulent rotating flow of liquid sodium exhibits self-excited dynamo action, polarity reversals, and excursions. In the present language, these may be interpreted as emergent collective transitions of coarse-grained local dynamo elements, although in VKS, the influence of boundary conditions, especially soft-iron impellers, is known to be essential. Thus SSM is not an alternative to the low-dimensional mode descriptions of VKS, but may provide a mesoscopic viewpoint underlying them. Table 2 sketches a provisional extension of the same SSM language to other cosmic magnetic-activity systems and to laboratory dynamos.

## 10. Conclusions

We have proposed the Synchronized Spin Model as a mesoscopic nonequilibrium framework for black-hole accretion systems. The key claim is that a rotating magnetized accretion flow can be coarse-grained into interacting magnetic domains in a Taylor-column-like picture whose synchronization, partial synchronization, excursion, and reversal govern both the timing statistics and the magnetic topology of the flow.

The model yields three main conclusions. First, partial synchronization and amplitude modulation provide a natural route to pink-noise continua, rms–flux/Taylor-like scaling, and approximately log-normal variability of the demodulated envelope. Second, a small set of collective variables,

(87) $$(M,G,f_{\mathrm{open}},C_{\mathrm{ax}}),$$

organizes the main morphological channels: closed topology favors the corona, partial coherent reversal favors flares, open but weakly axial topology favors winds, and ordered axial open topology favors jets. Third, the hard/soft/intermediate cycle can be read as motion through magnetic state space, with the hard branch retaining stronger macro-order, the jet-line region marking rapid restructuring, and the soft branch suppressing steady jets because its global dipole-like order is weaker.

The broader significance of SSM is to add a compact state-theory layer between first-principles simulations and phenomenological observables. Its purpose is not to replace GRMHD or launching theory but to connect variability statistics, magnetic topology, and outflow morphology within one framework.

In this revised statistical reading, the continuous variability of black-hole systems is more naturally characterized by multiplicative or approximately log-normal fluctuations than by a generic power-law flux distribution. By contrast, power-law-like event statistics remain a useful effective language only for suitably defined event catalogs or reconnection hierarchies, and are most directly established in solar and stellar flare systems. This distinction clarifies how the SSM can accommodate both rms–flux/log-normal phenomenology and broader multiscale reconnection activity without conflating continuous light-curve statistics with event-by-event energy distributions. In this sense, the SSM provides a falsifiable mesoscopic framework for black-hole accretion systems, whose main value lies in connecting timing statistics, magnetic topology, and outflow morphology within a single reduced-state language.

## Figures and Tables

**Table 1:** Three-level SSM interpretation of the black-hole X-ray binary q-diagram [1,2,29,30].

No.	State	Observed Phenomenology	Three-Level SSM Picture	Typical Ordering	Consistency/ Limits	SSM Predictions
1	Hard state	Strong broad-band noise; high RMS; compact flat-spectrum jet; radio/IR/X-ray correlation.	Noisy but globally ordered. Net poloidal flux sustains a steady jet while micro-fluctuations remain strong.	*m* high; *s* med–high; |M| high; *H* building.	Jet-producing state with persistent variability; not full synchrony.	Mean polarization axis is stable with rapid swings.
2	HIMS (rise)	Disk moves inward; IR drops; radio becomes variable and optically thin.	Global order weakens; meso-clusters become fragile; system approaches large-scale reorganization.	*m* high; *s* high but unstable; |M| falling; *H* large.	Jet instability starts.	Non-stationarity increases in lags and polarization.
3	Jet line/SIMS	Fast ejecta; transient radio flare; RMS drop.	Rapid magnetic restructuring: reversal or strong excursion releases stored energy and drives discrete ejecta.	*m* high; *s* rapidly varying; *M* rapidly varying; |dM/dt| maximal; *H* released.	Best SSM match; full reversal is possible but not required.	Rapid polarization-angle swings; timing anomalies; knotty/two-sided ejecta; flare strength tracks |dM/dt| more than |M|.
4	Soft state	Thermal disk to ISCO; steady jet quenched; weak radio may persist early; non-thermal tail may remain.	Thermally stable but weakly dipolar. Local reconnection continues, yet no strong jet-supporting macro field survives.	*m* high; *s* low–med; *M* low; *H* relaxing.	Explains jet quenching without removing all magnetic activity.	Local flares and coronal tails remain possible, but a sustained compact jet is hard to maintain.
5	Return HIMS (decay)	Similar to upper HIMS, but without strong optically thin flare; radio recovers at lower luminosity.	Large-scale order is rebuilt gradually rather than explosively.	*m* high; *s* med; *M* rising; *H* still important.	Hysteresis follows naturally if *H* stores topology/free energy.	Rise and decay should differ in polarization, and radio/X-ray correlation at the same hardness.
6	Quiescence	Low luminosity; recessed disk; weak timing activity; UV/optical thermal component.	Weakly excited, weakly coupled state; only low-level micro-activity remains.	*m* low–med; *s* low; *M* low; *H* fading.	Qualitatively consistent; detailed quiescent flow is better modeled in RIAF/evaporation pictures.	

Abbreviations: ISCO, innermost stable circular orbit; IR, infrared.

**Table 2:** Provisional extension of the Synchronized Spin Model (SSM) to a broader range of cosmic magnetic-activity systems and to laboratory dynamos. This table is intended as a phenomenological unification map. Recurrent activity is common, but a strictly periodic global cycle analogous to the solar cycle is not established in all systems.

System	Corona/Magnetosphere	Flare/Burst	Jet/CME/Ejecta	Activity Cycle or Recurrent Change	1/*f*-like Variability	Main Driver in SSM Language
XRB (BH) [1,2,7,15]	hot corona (∼100 keV)	X-ray flare	relativistic jet	hard/soft cycle; hysteretic state transitions	strong	MRI-driven disk dynamo + multiscale reconnection
AGN [4,5,18,19,20]	X-ray corona	X-ray flare	relativistic jet/disk wind	changing-look transition; long-term state change	strong	strong disk dynamo + flux accumulation/reorganization
NS (magnetar/high-B NS)	magnetosphere	X-ray/γ-ray flare, burst storm	relativistic outflow/plasmoid-like ejecta	recurrent outbursts; glitches may accompany activity, but no universal cycle	present in some cases	crust–core stress + magnetic twist/reconnection
Protostar/YSO	stellar/star–disk corona	X-ray flare; accretion burst	protostellar jet/outflow	episodic accretion and outflow recurrence; no generic solar-like cycle	reported in some data	star–disk dynamo + episodic accretion + reconnection
Sun	solar corona	solar flare	CME	11-year activity cycle (22-year cycle)	clear	αΩ dynamo + flux transport + reconnection
Active stars [16,37,49]	stellar corona	stellar flare/superflares	stellar CME candidates	magnetic activity cycles in many stars	reported in some cases	stellar dynamo
Earth	magnetosphere	substorm	plasmoid tail jet/bursty bulk flow	secular variation, excursions, and reversals; no stable periodic cycle	present in geomagnetic indices	internal geodynamo + solar-wind coupling
Jupiter and giant planets [50,51]	magnetosphere	auroral burst/injection event	magnetotail jet/plasmoid ejection	rotation-modulated and secular variability; no solar-like cycle	broad-band/ULF fluctuations reported	internal dynamo + rapid rotation + external plasma loading/solar-wind forcing
Laboratory dynamo (VKS) [46,47,48,52]	nonequilibrium conducting-fluid volume	magnetic burst/excursion	impulsive global field reorganization	irregular polarity reversals, excursions, and stationary/oscillatory dynamo regimes	reported in magnetic induction time series	turbulent liquid-sodium dynamo + mode competition + boundary-condition effects (especially soft-iron impellers)

## Data Availability

The raw data supporting the conclusions of this article will be made available by the authors on request.
